# Comparison of Preschooler Verbal and Graphic Symbol Production Across Different Syntactic Structures

**DOI:** 10.3389/fpsyg.2021.702652

**Published:** 2021-11-25

**Authors:** Gat Savaldi-Harussi, Leah Fostick

**Affiliations:** Department of Communication Disorders, Ariel University, Ariel, Israel

**Keywords:** expressive use of graphic symbols, clause construction, augmentative and alternative communication, graphic symbol modality, native speakers, transitive and non-transitive verbs, language representation

## Abstract

The present study focuses on the impact of graphic symbols used in Augmentative and Alternative Communication (AAC) on clause construction. It is not yet well-understood to what extent communication produced via graphic symbols differs from verbal production. This study attempts shed light on the impact of the graphic symbol modality on message construction beyond individual differences, language knowledge, and language-specific patterns by providing a direct comparison between children’s verbal and graphic symbol production. Nineteen typically developing Hebrew-speaking children aged 4–5 years were presented with 16 short videos of actions and were asked to express what they saw verbally and by choosing among graphic symbols displayed on an iPad communication board. The 570 clauses produced by the children were coded and analyzed. A significant difference was found in favor of verbal speech across different syntactic structures in terms of utilization of the target lexicon, syntactic complexity, and expected target word order. These results are consistent with the existing literature for English. Implications for AAC practices are discussed, highlighting the notion that using graphic symbols to represent spoken language may not reflect actual linguistic knowledge and that adequate, explicit instruction is necessary for graphic representation of more complex linguistic structures.

## Introduction

A diverse population uses Augmentative and Alternative Communication (AAC) services, including children and adults with developmental and acquired disabilities whose ability to use natural speech is affected by severe speech or language difficulties (Smith, 1996, 2006; Binger and Light, 2008). AAC is an area of clinical practice that provides tools and techniques to supplement or replace speech, including the use of unaided communication (e.g., gestures, facial expression) and “aided communication” such as graphic symbols displayed on communication devices to represent spoken language (American Speech-Language-Hearing Association, 2020). AAC is often utilized within the context of multimodal communication, which involves selecting the preferred mode of communication (e.g., aided or unaided) that allows the most efficient self-expression. Among those who use AAC and are not yet literate, graphic symbols are the primary communication modality (Von Tetzchner and Grove, 2003).

In the typical, natural course of communication development, children in many cultures are thought to extend their spoken communication by developing external, visually based symbols to communicate information (e.g., numbers, alphabet letters, and pictorial signs). Psycholinguistics research has found that external symbols accelerate the communication of knowledge and play a crucial role in enhancing human intelligence (Lee and Karmiloff-Smith, 1996; Mavrou et al., 2013). The automatic tendency to use multimodal communication, such as a combination of natural speech along with manual signs and external symbols, has become a principal practice in AAC (Loncke, 2014).

Symbolization, a fundamental component of AAC, is a vast area of research whose definition is pertinent to this study. Symbolization includes two elements: a signifier and a signified. The signifier is defined as something that stands for the signified: be it an idea, person, or object. In cognitive psychology, various terms such as “symbol,” “sign,” “icon,” and “notation” are used to refer to signifiers (Lee and Karmiloff-Smith, 1996). Peirce’s theory of signs (1965–1966) suggests that while a sign is the smallest unit of meaning, symbolic signs are arbitrary (e.g., words), and thus the relation between signifier and signified is based on convention. In contrast, iconic signs (e.g., pictures) refer to signifiers that resemble the signified, and indexical signs are those in which the relation between signifier and signifies is based on cause and effect (e.g., smoke and fire) (Atkin, 2014).

In the AAC field, the term “graphic symbol” or “symbol” is used for pictures and graphic representations that are signifiers of ideas the person wishes to convey (Loncke, 2014; Pampoulou and Fuller, 2020). “Graphic symbols” can be part of a symbol system with rules about building the pictures (e.g., Bliss words, Blissymbols) or a symbol set without internal principles for symbol formation (Fuller et al., 1992; Loncke, 2014; Pampoulou and Fuller, 2020). Graphic symbols often take the form of line drawings such as Picture Communication Symbols (PCS), SymbolStix©^1^ (Clark, 1997), or Widgit©^2^ (Kennedy, 2004) symbols. A set of graphic symbols aiming to represent the spoken language-for the purpose of communication- may include iconic, symbolic, and indexical signs depending on the target referent.

The ability to decode visual forms (such as graphic symbols) depends to a large extent on biological, cognitive, and cultural factors (Lee and Karmiloff-Smith, 1996). Children begin to understand that a symbol (signifier) stands for something else at around the age of two and, by the age of 6–7 years, understand most conventional notation systems (visual forms). That said, there is variation in the pace of development across different visual representations (e.g., drawing vs. written language) (Lee and Karmiloff-Smith, 1996). Indeed, an individual who uses graphic symbols to communicate must have well-developed internal visual representational skills and understand that a symbol is an object by itself and at the same time refers to something else (Loncke, 2014).

The term *iconicity* refers to the representation value of the graphic symbol’s *image*, ranging from transparent (i.e., the graphic symbol displays the word’s exact meaning, such as a picture of a house to represent the word “house”) to translucent (i.e., an indirect relationship between the symbol and word, such as a horizontal line and a dot on top to represent the word “on”) (Loncke, 2014). Graphic symbols in AAC are designed to be as transparent as possible, visually representing the target referent.

Considering the characteristics and constraints of visual forms such as the graphic symbols used in AAC, one of the big questions in the AAC field is to what extent production via graphic symbols differs from verbal production. Therefore, this study provides a direct comparison between children’s verbal and graphic symbol production after they watch short, silent videos depicting a boy engaging in different actions.

Picture Communication Symbols graphic symbols (signifiers) were selected for this study because of their easy learnability. However, it is important to note that this set of graphic symbols include symbols representing both concrete and abstract referents, thus ranging from transparent to translucent. For example, concrete referents (e.g., a symbol of a boy representing a boy) are considered to have high transparency and are more iconic, as often one can “look through” the symbol (signifier) and easily extract its meaning (Loncke, 2014). In contrast, a graphic symbol is considered translucent when the relationship to the meaning becomes clear only after revealing or learning its meaning. Consequently, representing certain linguistic features (e.g., prepositions, connectors) via graphic symbols may be challenging and not reflect one’s actual linguistic mental representation.

### Characteristics of the Graphic Symbol Modality

Smith (2006) describes the characteristics of the graphic symbol modality in comparison to natural spoken language. One noted difference, as described above, is the connection between the symbol and its referent; while a spoken word consists of arbitrary sounds that represent a specific referent and therefore the connection between the spoken word and its referent is arbitrary, a graphic symbol is designed to be iconic.

Another difference between graphic symbols and spoken language is related to segmental features. Words are composed of a limited set of meaningless segments (phonemes) that can represent infinite meaningful morphemes and that can be combined to create new meanings, resulting in a simultaneously economical and productive language system. In contrast, graphic symbols (e.g., PCS, SymbolStix) represent a finite set of symbols that cannot be divided into subcomponents and create new meanings. Therefore, the set of graphic symbols is not characterized by the same productivity as oral language. A final difference is that oral language is produced by the human body via a process of sorting words and linguistic structures from the mental lexicon, while graphic symbols are represented externally and visually by a finite set of symbols designed and organized by someone else.

One significant challenge for those who use graphic symbols is the mismatch between spoken language input and graphic symbol output (Trudeau et al., 2007). Since the goal of graphic symbol communication is the recording of verbal-based messages into graphic symbols, the output is expected to mirror spoken language structural properties. Therefore, the individual using graphic symbols needs to make a connection between the two modalities in a task of “translation” in which they must switch from one modality to the other by recruiting metalinguistic abilities (Sutton et al., 2002, 2020; Smith, 2006; Trudeau et al., 2007).

Moreover, representing linguistic structures in graphic symbols is also challenging because grammatical category boundaries of spoken utterances may be unclear when transmitting the message into graphic symbols. For instance, when representing verbs via graphic symbols, information regarding the predicate and its arguments may all appear in a single graphic symbol: the action (verb) and its agent (pronoun) may be displayed simultaneously in a single static graphic symbol. An example of this is the verb SIT which is represented by PCS as a line drawing of a person sitting on a chair, viewed in profile. This graphic symbol includes the agent and the object sat upon, and simultaneously represents the action of sitting, the agent, and the object (Smith, 2015). Therefore, rather than selecting three different graphic symbols that represent the three content words (square brackets in the example below) of its eight morphemes, the *single* symbol, in essence, represents the following full sentence:


$$\begin{array}{ccccc} \mathrm{The person} & \mathrm{is} & \mathrm{sitting} & \mathrm{on a} & \mathrm{chair}.\\ \mathrm{Art}\,\left[\mathrm{NOUN}\right] & \mathrm{Copula} & \left[\mathrm{VERB}+\mathrm{ING}\right] & \mathrm{PrepArt} & \left[\mathrm{NOUN}\right] \end{array}$$

Similarly, graphic symbols for verbs THROW and PUSH also depict the agent who performs the action, the action itself, and the object of the verb (Sutton et al., 2002).

In recent decades, studies in the field of AAC have attempted to explore the characteristics and constraints of the graphic symbol modality. One related question is to what extent the patterns observed in the word order, syntactic complexity, and lexicon of individuals who use graphic symbols differ from those of typically developing individuals using spoken language, and whether the observed differences are due to individual differences or due to the modality itself (Trudeau et al., 2007, 2010a,b; Savaldi-Harussi et al., 2019; Sutton et al., 2020).

### Graphic Symbol and Language Outcomes

Analyzing linguistic structures produced via graphic symbols requires defining the *unit of analysis* based on the characteristics of aided communication (Müller and Soto, 2002; Kovacs and Hill, 2017). In psycholinguistics, an utterance is defined as behavioral stretches of oral output and a clause is defined as “any unit that contains a unified predicate… (that is) a predicate that expresses a single situation” (Berman and Slobin, 1994). For the purposes of this study, the term *clause construction* refers to construction via graphic symbols. Due to the co-constructed interaction feature of aided conversation, in which the message is co-constructed with the adult’s scaffolding; researchers often study utterances produced during conversation turns rather than utterances, *per se*; in such a case, the unit of analysis would be the message constructed via graphic symbols without the adult intervening (Savaldi-Harussi and Soto, 2016).

Studies conducted in English language environments have found that children who use AAC have difficulties in tasks that evaluate morpho-syntactic knowledge. Such tasks require judgment about whether a target sentence sounds correct or not (e.g., “Tomorrow they walked”). Children who use AAC have demonstrated difficulties identifying and marking mandatory inflections, manifested in nouns or verbs used without following grammatical standards of verbal and nominal inflections (e.g., suffixes “-ed,” “-s,” and “-ing” for verbs, and plural “-s” for nouns) and the nominative case (e.g., possessive “’s”), resulting in short construction (e.g., “girl eat banana” instead of “THE girl IS eatING A banana”) (Sutton and Gallagher, 1993; Redmond and Johnston, 2001; Blockberger and Johnston, 2003; Savaldi-Harussi and Soto, 2018, Savaldi-Harussi et al., 2019).

Four main patterns have been identified in the expressive language of individuals who use graphic symbols: (1) dominance of utterances (messages) with a single symbol; (2) perseverance of simple structures; (3) changes in word order; and (4) grammatical errors (Smith, 2015). Although output of a single symbol is widely reported (Sutton et al., 2002; Savaldi-Harussi and Soto, 2018), children who use AAC can generate multi-symbol utterances (messages) in which simple constructions of subject–verb–object (SVO) are the common structure (Sutton et al., 2002; Savaldi-Harussi et al., 2019). However, these simple constructions were not found to follow the typical word order of the common clause structure in English consisting of SVO (e.g., MAN DRIVE CAR). Instead, these children were found to use the following structures instead: Subject-Object-Verb (MAN CAR DRIVE), Verb-Subject-Object (DRIVE MAN CAR), or Object-Verb-Subject (CAR DRIVE MAN). When forming complex construction, graphic symbol users tend to change word order in multiple positions: GIRL TREE HELP NEST CLIMB BOY (instead of “the girls help the boy climb a tree to get a nest”; Soto, 1997). Lastly, constructions via graphic symbols have been reported to include key symbols but lack grammatical markers such as auxiliaries, articles, prepositions, and suffixes-even though grammatical markers are available in the communication devices-resulting in ungrammatical structures (Binger and Light, 2008; Sutton et al., 2010).

Indeed, morpho-syntactic differences between the graphic symbol modality and spoken language have been observed in children (Blockberger and Johnston, 2003), adolescents (Redmond and Johnston, 2001), and adults (Sutton and Gallagher, 1993) with typical and atypical language production. Sutton et al. (2010) observed how 30 preschool children transferred SVO structures to graphic symbols, reporting that at least one core element (subject, verb, or object) was missing in more than 50% of the expressions produced, with verbs accounting for 78% of the omissions. One possible explanation of this finding is a relatively low level of iconicity of target verbs in graphic symbols, making it developmentally difficult for young children to represent them (Von Tetzchner and Grove, 2003).

Another possible explanation for atypical linguistic patterns in messages constructed in English via graphic symbols is a lack of attention to linguistic markers that are perceptually less salient to AAC users. Less attention is paid to aspects of language that have little semantic value due to insufficient learning and practicing of morphological rules among this population (Blockberger and Johnston, 2003). For instance, parts of speech in English (e.g., nouns, verbs, and adjectives) often appear as bare stems or as free morphemes, and inflections only play a minor role in the relationship between parts of a sentence, while word order provides the critical information (Dromi et al., 1993). Moreover, adding morphological markers to lexical stems via graphic symbols requires cognitive-linguistic effort, memory, and physical effort (Loncke, 2014); thus, short sentences are an effective strategy to enhance the communication pace. This explanation strengthens the notion of particular challenges when mapping spoken language structures onto graphic symbols and puts the modality as the source of the atypical structure, beyond the communication difficulties of those who use it.

To explore whether the level of attention to grammatical markers impacts the morpho-syntactic differences between graphic symbol expression and spoken language, researchers suggested conducting cross-linguistic studies in languages that include grammatical morphology with greater perceptual salience than English (Blockberger and Johnston, 2003; Smith, 2015). Such cross-linguistic studies can shed light on the impact of the graphic symbol modality on message construction beyond individual differences, language knowledge, and language-specific patterns. This is the purpose of the current study.

### Contrasting English and Hebrew

Hebrew is a Semitic language with rich morphology. In contrast to English, in which nouns, verbs, and adjectives are often used as bare stems, and are formed by affixation (e.g., dance + er → dancer), zero-conversion (e.g., work–to work), and compounds (e.g., high-school, daycare) (Clark and Berman, 1987; Dromi et al., 1993; Berman et al., 2009), formation of verbs and certain adjective and nouns in Hebrew occur through integrating a consonantal root (e.g., R-Q-D) into a pattern (e.g., CaCCan) to form the word (RaQDan = dancer). The root conveys the core meaning of a word (R-Q-D represents “dance”) and often consists of three consonants. Words are also inflectionally marked for number and gender (in Hebrew, animate and inanimate nouns are also marked for number and gender). Verbs are also inflected for tense and need to agree with their subject noun in number, gender, and person: present tense forms are marked for number and gender, whereas past tense forms are marked for person (first, second, and third) as well as number and gender. Moreover, verbs have a special form for the imperative and infinitive (Berman, 1985; Dromi et al., 1993). The least inflected form of Hebrew verb is the masculine singular in present tense (e.g., “moxer” = sell) and the third-person masculine singular in past tense (e.g., “maxar” = sold) that have no prefixes or suffixes. These forms in Hebrew are treated as basic although they are inflected (Dromi et al., 1993). English and Hebrew also differ in their functional categories. English has a definite as well as an indefinite article (*the* and *a(n)*, respectively) while Hebrew marks only the definite article and has an overt accusative marker *et* before a definite object, which is not marked in English.

Subject–verb–object structure is the canonical form in English in which subject-first forms predominate. This especially occurs in utterances involving two nouns and a verb in which the agent is animate and the patient inanimate (Slobin and Bever, 1982). In Hebrew, the word order of a sentence including a verb resembles the English word order SVO (Glinert, 2017). Slobin and Bever (1982) found that the average age for children to use word order strategy is around 3:6, and children are attuned to these canonical sentences.

While graphic symbol use has been researched in English, little research has been done in Hebrew (Vinder, 2016; Mano-Lerman, 2017). Consequently, the aim of current study is twofold: (1) to compare the constructions produced via graphic symbols to those produced verbally across different syntactic structure: subject verb (SV), SVO, and two coordinated clause SV[and]SV, and (2) to compare these constructions by focusing on differences in lexicon, syntactic complexity, and word order across different syntactic levels. This study focused on typically developed (TD) Hebrew-speaking children aged 4–5 years, as at this age children are expected to be at the late linguistic stage in which they acquire coordination structures (Dromi et al., 1993) and develop good internal visual representation. Early literacy skills, such as letter knowledge and print concept, also emerge at this age (Treiman et al., 2007).

The goal of this paper, therefore, is to answer the following questions:

(1) Are there production differences in semantic-syntactic representation of clause structure (lexicon, syntactic complexity, and word order) in Hebrew when using graphic symbols vs. speech?
(2) Are there production differences in clause structure in Hebrew when using different syntactic structures: SV, SVO, [SV] and [SV] across modalities (verbal vs. graphic symbol)?

## Materials and Methods

### Participants and Setting

Nineteen TD preschoolers between age 3:8 and 5:01 (years:months) participated in the final cohort (9 girls/10 boys; *M*_*age*_ = 4:03, *SD*_*age*_ = 0.45). The original sample included 20 participants, but one was excluded from the study once it was determined that they were receiving speech and language therapy. To qualify for inclusion, children had to meet the following criteria:

(a) be a native speaker of Hebrew;
(b) attained a Sentence Repetition score within 1.5 standard deviations [using a subtest from *The Goralnik Screening Test for Hebrew* (Goralnik, 1995). This test, also known as “sentence recall and sentence imitation,” includes different morpho-syntactic structures and serves as a reliable screening task to identify specific language impairments (Theodorou et al., 2017)]; and
(c) have hearing, visual, neurological, linguistic, and communicative development with the normal range based on parental report. No record of speech and language impairment.

The study was carried out at each child’s home in a quiet room. A familiar adult was permitted to join the session and instructed to observe without participating. Third-year speech-language pathology students administered tests under the supervision of the first author. The study was approved by the IRB University (AU-HEA-GH-20190130-1) and was conducted in accordance with appropriate ethical standards.

### Materials

#### Videos Displayed on Interactive Board Game

Sixteen short videos (*M* = 5.05 s, *SD* = 3.2 s) of a young boy performing different actions were displayed on a laptop screen within a fun, interactive path board game using Power Point slides. These videos provided only visual representation with no verbal input. The path board included 15 interactive steps organized on a screen with numbers from 1 to 15. When a participant clicked on a step, a video appeared; after watching the video, the participant was asked to describe verbally, and then via graphic symbols, what had just been seen. The videos were designed to elicit target utterances of various syntactic structures described below. To elicit two coordinated clauses, two videos were displayed next to each other with a plus sign (+) between them. Each slide had a button on the right that navigated back to the home board game. Two different orders of videos were used to control the effect of fatigue on the last sentences elicited; the differently ordered videos were randomly assigned to each participant.

#### Verbs and Syntactic Structures Probe

The target structures were utterances with one clause or two clauses. For utterances with one clause, the target structures were SV and SVO; for utterances with two clauses, the target structure was SV[and]SV. A total of 15 utterances were targeted which included five utterances for each syntactic structure: SV, SVO, and two coordinated clauses (using two SV clauses and the Hebrew coordinator VE [and]). The targets SV, SVO, and SV[and] SV are depicted in Table 1. For the SV structure, five non-transitive verbs were selected: jump (*kofets*), laugh (*tzoxek*), dance (*roked*), sleep (*yashen*), and shower (*mitkaleax*). For the SVO structure, five transitive verbs were selected as follows: open (*poteax*); throw (*zorek*), wear (*lovesh*), hold (*maxzik*), and hug (*mexabeck*). These verbs were selected because they emerge early in children’s lexicons. Subject-Verb agreement was singular (SG) and masculine (MS) in grammatical number and gender (*kofets* = jumpSG.MS.Present), which is the basic form in Hebrew, and was targeted by presenting one boy (agent) performing different actions in the videos.

**TABLE 1:** Target constructions (SV, SVO, and Coordination Clauses).

Subject verb	Subject verb object	Coordination sentence
The *boy* is *jumping*	The *boy* is *opening* the *door*	The *boy* is *laughing*, and the *boy* is *crying*
The *boy* is *dancing*	The *boy* is *throwing* the *ball*	The *boy* is *showering*, and the *boy* is *drying*
The *boy* is *sleeping*	The *boy* is *wearing* a *shirt*	The *boy* is *swinging*, and the *boy* is *sliding*
The *boy* is *showering*	The *boy* is *hugging* the *bear*	The *boy* is *playing*, and the *boy* is *jumping*
The *boy* is *laughing*	The *boy* is *holding/reading* a *book*	The *boy* is *walking*, and the *boy* is *sleeping*
		

#### Grid Symbols Display

Twenty-three graphic symbols were displayed on a communication board using the AAC application GRID© on an iPad. Twenty-two colored PCS symbols (Mayer-Johnson, 1981–2011) and one letter symbol were displayed on the board as follows: 16 verbs (jump, dance, sleep, showering, laugh, open, throw, wear, hug, slide, swing, hold, play, cry, walk, and dry), six nouns, and one letter for the word *and*. The Hebrew conjunction word VE (*and*) is a bound morpheme attached as a prefix to the words it connects, for example: “John and Mary” would be “John ve Mary.” The parts of speech were organized on the board from right to left, the direction of writing in Hebrew, with the pronoun BOY, the agent of all the action, in the right column and the verbs in the left columns. The background of the symbols follows the Fitzburg Key (Fitzgerald, 1969) color codes for distinguishing different parts of speech: green for verbs and yellow for nouns. The board included a message window that visually presented the constructed symbols and voice output in the form of digitized speech (“Matan”). The user can activate the message window and receive auditory feedback on the constructed message. Each symbol/button also has a voice output that serves as auditory feedback. The children could modify the message by deleting a single symbol or the whole message and indicate when the constructed message was done.

### Procedure

Prior to the start of the study, a verbal explanation was provided to the guardian of each of the participants about the purpose and procedures of the research, and they were also given a detailed written explanation via the consent form. Those who wished to take part in the research provided signed informed consent on behalf of their children to participate in the study and filled out a questionnaire about their child’s personal, developmental, and demographic details. As a part of the study, a screening test was conducted for each participant using the sentence repetition subtest from the Goralnik assessment tool. Next, children were trained to use the AAC (see section “Familiarization and Training” for further details). During the experimental phase, the children were given the following instruction about the interactive game board: “Watch a video and say aloud what you see. Then, say it with the symbols on the board.” All participants’ productions, both verbal and graphically symbolic, were documented. All meetings took place in a quiet room and the presence of an adult familiar to the participants was allowed in order to achieve maximum cooperation on the part of the participants. Each session lasted about 45 min and was conducted by university students from the Communication Disorders department. Participants’ results were assessed by at least two evaluators. All experimental sessions were carried out by research assistants who were speech-language pathology students under the supervision of the first author.

#### Familiarization and Training

Before the experiment was conducted, the children were trained to use the AAC board. Research assistants presented the symbols that appear on the AAC board. The familiarization phase included two steps, as follows: first, the participant was asked to name each symbol and then to click on the symbol to receive the auditory feedback. The children’s naming of the symbols was documented. Then, the child was asked to construct six structures (two SV, two SVO, and two coordinated clauses) with the graphic symbols that were different from the target sentences. Following each structure, a research assistant modeled how to construct it correctly with the displayed graphic symbols. The graphic symbols used during training were the same as those used for the experimental phase, but the combinations of the verbs and the syntactic structure were different. For example, the verb “walk” was modeled with the syntactic structure SV in “The boy is walking,” by selecting the target symbols “BOY, WALK,” but during the trial it appeared within two coordinated clauses ([SV] AND [SV]) of “The boy is walking, and the boy is sleeping.”

The naming of the graphic symbols in the training phase was coded to identify symbol transparency. The coding for naming was as follows: three points for a correct response, two points for a semantically close response, and one point for saying an unrelated word or phrase. The transparency level was calculated as the average score of each symbol. Nouns were fully transparent while the verbs varied in their transparency level. We also documented if the participants were familiar with the letter VAV (yes/no). Only 4 (21%) out of the 19 participants identified the letter symbol VAV when it was first introduced.

#### Scoring and Reliability

All children’s production of verbal and graphic symbols (570 clauses) were coded and analyzed in three aspects: lexicon, syntactic complexity, and word order, as described in Table 2.

**TABLE 2:** Metric score for word order, syntactic complexity, and lexicon.

Scoring	Word order	Syntactic complexity	Lexicon
0	Did not maintain proper word order	Arguments only (noun)	Replaced or omitted more than one content word from the target sentence
1	Maintained proper word order	Verb only without arguments	Replaced or omitted one content word from the target sentence
2		Verb + argument [SV]	Retained all target content words
3		Verb + 2 arguments [SVO]	
6		[SV] AND [SV]	

For evaluating the children’s lexicon, the maximum score was two points when all target content words were used in the production, one point was given when one content word was omitted or replaced, and zero points given for more than one error.

Syntactic complexity refers to the difference between [SV] and [SVO] (having a complement to the verb) or between [SV] and [SV and SV] (a simple clause or a coordinate clause). For syntactic complexity, based on the metric adapted from Savaldi-Harussi et al. (2019) and modified for the current study, each content word (verb or noun) received one point while the connection word “*and*” (indicating more complex production) gained additional two points. Therefore, a maximum syntax score of 6 was allocated for the two coordinated clauses [SV] AND [SV].

Word order refers to the order of the constituents within the different structures. For example, for the structure SVO, children might answer SV, VSO, or OSV. Only the last two are counted for word order errors, while all three will be counted as errors when comparing the different structures. The maximum score for the word order component was one point when the content words followed the canonical order of Hebrew sentence structure. For example, for the target sentence “The boy (NOUN1) is hugging (VERB) the bear (NOUN2),” a participant may have produced the following responses (a) verbally and (b) in graphic symbols:

(a) *Hugging* (VERB) *bear* (NOUN2)
(b) *BOY* (NOUN1) *BEAR* (NOUN2)

Scoring of the above example would have been done as follows. In (a), for the verbal production consisting of [VERB, NOUN2], only one point would have been given for the lexicon component as the participant omitted one content word (NOUN1), two points would be allocated for the syntactic complexity as one verb and one noun were used, and one point would have been allocated for the word order as the verb-object order had been maintained. For the constructions produced via graphic symbols in (b), [NOUN1, NOUN2], one point would have been allocated for the lexicon component as one content word [VERB] was omitted, zero points given for syntactic complexity as a verb was not used, and one point given for the word order as the argument order of the SVO structure was maintained.

Two communication disorders students coded the 570 constructions produced by the children. Although the analysis was straightforward, unclear cases were discussed and resolved with the first author. For example, the verb *wear* can take two forms in Hebrew to express the meaning of “the boy is wearing”: the first, a transitive verb “lovesh,” requires an object, while the other form, an intransitive verb *hitlabesh* (literally “dressed himself”), does not require an object. Therefore, a syntactic score of two points was allocated to both forms.

### Analysis

For statistical analysis purposes, the scores of lexicon, syntactic complexity, and word order were converted to percentages (raw scores divided by maximum score for each sematic-syntactic representation) as presented in Table 2. A two-way repeated measures ANOVA was performed, with symbol modality (verbal, graphic), syntactic structure (SV, SVO, and SV + SV), and semantic-syntactic representation (lexicon, syntactic complexity, word order) as within-subjects variables. *Post hoc* analyses were performed using least significant differences (LSD) and *t*-tests.

## Results

Table 3 presents the mean score of the 19 participants for each semantic-syntactic component (lexicon, syntactic complexity, word order) across syntactic structure (SV, SVO, [SV] + [SV]) and modality (verbal, graphic symbol). The maximum score for each semantic-syntactic component, the mean score (*M*), and the percentage (%) out of the maximum score, are all presented.

**TABLE 3:** Sematic-syntactic scores across syntactic structure and modalities.

Syntactic structure		SV			SVO			[SV] AND [SV]		
Sematic-syntactic		Lexicon	Syntactic complexity	Word order	Lexicon	Syntactic complexity	Word order	Lexicon	Syntactic complexity	Word order
Max score		2	2	1	2	3	1	2	6	1
Modality	Verbal *M* (%)	1.38 (68.95)	1.44 (72.11)	0.48 (48.42)	1.11 (55.26)	2.18 (72.63)	0.72 (71.58)	1.45 (72.63)	4.61 (76.84)	0.88 (88.42)
	GS *M* (%)	1.25 (62.63)	1.24 (62.63)	0.24 (24.21)	0.66 (33.16)	1.11 (36.84)	0.28 (28.42)	1.28 (64.21)	2.95 (49.12)	0.33 (32.63)

*GS = Graphic symbol.*

Significant main effects were found for symbol modality, *F*(1,17) = 17.695, *p* = 0.001, partial η^2^ = 0.510, syntactic structure, *F*(2,34) = 9.935, *p* = 0.000, partial η^2^ = 0.369, and semantic-syntactic representation, *F*(2,34) = 16.193, *p* = 0.000, partial η^2^ = 0.488. No effect was found for gender, *F*(1,17) = 0.358, *p* = 0.558, partial η^2^ = 0.021. In general, verbal scores were higher (*M* = 69.992%, *SD* = 23.865) than when graphic symbols were used (*M* = 43.704%, *SD* = 30.443). Higher scores were obtained for SV (*M* = 56.611%, *SD* = 24.336) and SV + SV (*M* = 64.160%, *SD* = 23.569) structures than SVO structures (*M* = 49.772%, *SD* = 27.173, *LSD* = 6.840, *SE* = 2.720, *p* = 0.022 and *LSD* = 14.389, *SE* = 3.246, *p* < 0.001, respectively). Finally, higher scores were observed for lexicon (*M* = 59.602, *SD* = 23.002) and syntactic complexity (*M* = 61.719, *SD* = 19.271) than for word order (*M* = 49.222, *SD* = 29.872, *LSD* = 10.380, *SE* = 2.494, *p* = 0.001 and *LSD* = 12.497, *SE* = 2.911, *p* < 0.001, respectively).

**FIGURE 1:**
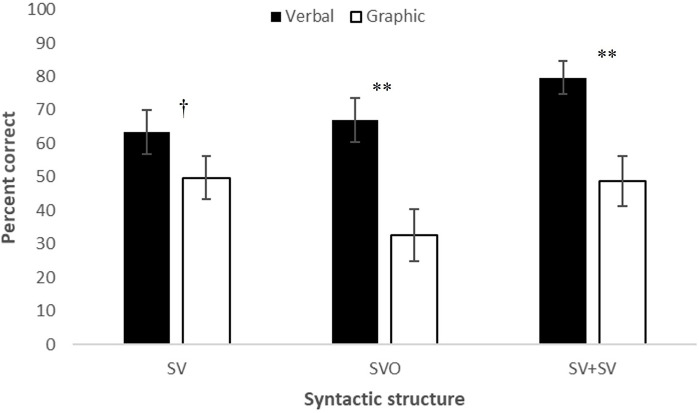
Verbal and graphic scores for different syntactic structures. ^†^*p* = 0.05; ^**^p < 0.001.

**FIGURE 2:**
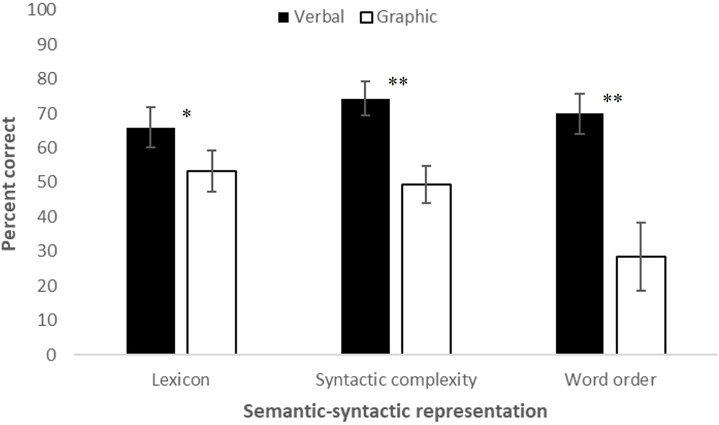
Verbal and graphic scores for the different sematic-syntactic representation. *p < 0.05; ^**^p < 0.001.

Interactions of Modality × Syntactic Structure, *F*(2,34) = 10.305, *p* = 0.000, partial η^2^ = 0.377, Modality × Semantic-Syntactic Representation, *F*(2,34) = 24.699, *p* < 0.001, partial η^2^ = 0.592, and Syntactic Structure (Type of Clause) × Semantic-Syntactic Representation, *F*(4,68) = 29.914, *p* < 0.001, partial η^2^ = 0.638, were found, as well as a Modality × Type of Clause × Complexity interaction, *F*(4,68) = 4.904, *p* = 0.002, partial η^2^ = 0.224. Figure 1 presents scores for each type of syntactic structure when produced verbally and via graphic symbols; Figure 2 presents scores for each semantic-syntactic component when produced verbally and via symbols. As can be observed from both figures, although the interactions were significant, verbal production gained higher scores than graphic production for all syntactic structures (SV: *t*(18) = 2.059, *p* = 0.054; SVO: *t*(18) = 4.440, *p* < 0.001; SV + SV: *t*(18) = 4.701, *p* < 0.001) and all semantic-syntactic representations (lexicon: *t*(18) = 2.294, *p* = 0.034; syntactic complexity: *t*(18) = 4.432, *p* < 0.001; word order: *t*(18) = 4.759, *p* < 0.001).

**FIGURE 3:**
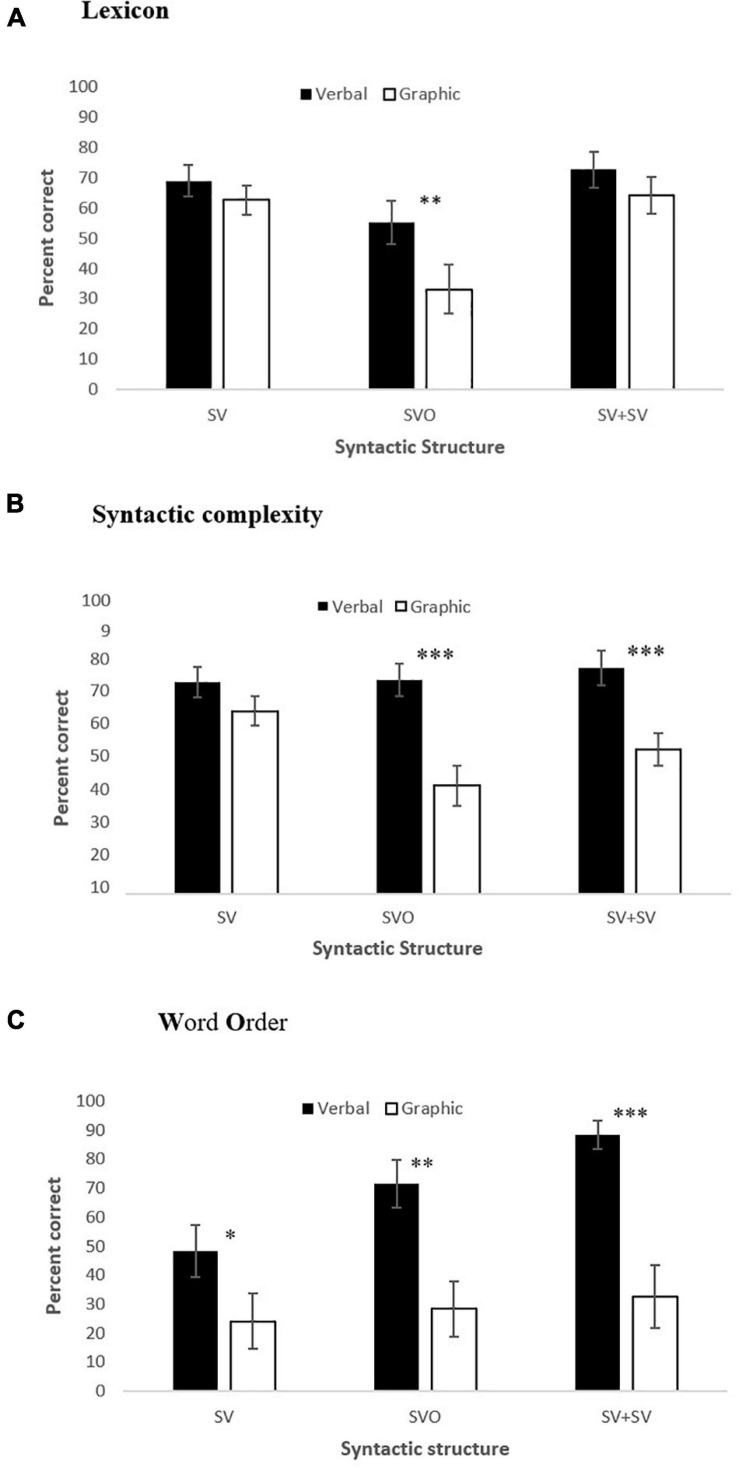
Scores for SV, SVO, and SV + SV clauses when produced in verbal and graphic symbols, separately for **(A)** lexicon, **(B)** syntactic complexity, and **(C)** word order. **(A)** ^**^p < 0.01. **(B)** ^***^p < 0.001. **(C)** *p < 0.05; ^**^p < 0.01; ^***^p < 0.001.

Figure 3 presents scores for each syntactic structure (SV, SVO, SV + SV) when produced in verbal and graphic symbols, separately for: (Figure 3A) lexicon, (Figure 3B) syntactic complexity, and (Figure 3C) word order. As can be observed, when divided between semantic-syntactic representation, verbal production earned higher scores than graphic symbols in almost all, but not all, conditions. Verbal production elicited higher lexicon scores in SVO clauses (*t*(18) = 3.200, *p* = 0.005), but not in SV (*t*(18) = 1.189, *p* = 0.250) or SV + SV clauses (*t*(18) = 1.455, *p* = 0.163). Verbal production also resulted in higher syntactic scores in SVO (*t*(18) = 4.720, *p* < 0.001) and SV + SV (*t*(18) = 4.443, *p* < 0.001) clauses, but not in SV (*t*(18) = 1.994, *p* = 0.062) clauses. Finally, verbal production elicited higher word order scores in all types of clauses (SV: *t*(18) = 2.516, *p* = 0.022; SVO: *t*(18) = 4.194, *p* = 0.001; SV + SV: *t*(18) = 5.463, *p* < 0.001) compared to graphic symbols.

## Discussion

The goal of this study was to shed light on the impact of the graphic symbol modality on clause construction in Hebrew, a Semitic language with rich morphology, and to explore the relationship between verbal production and the graphic symbol modality. Specifically, this study was designed to examine the effect of the graphic symbol modality on the semantic-syntactic representation (lexicon, syntactic complexity, word order) of different syntactic structures presented in SV, SVO, and coordinated clauses ([SV] AND [SV]) among young typically developing children aged 4–5 years who speak Hebrew.

In general, the young, typically developing children who participated in this study earned higher verbal production scores than graphic production scores for all syntactic structures (SV, SVO, and SV + SV) and for all semantic-syntactic representations (lexicon, syntactic complexity, and word order). The results of this study are consistent with the existing literature in the English language regarding word order and syntactic structure. For example, Smith (1996) found that, among five typically developing preschoolers aged 3:5–4:7, even after 10 weeks of learning and practicing the production of sentences using a board that included 53 PCS, the differences between verbal and AAC production were significant; production using graphic symbols was mostly single-image expression. In another study (Sutton and Morford, 1998) 32 typically developing children aged 5:9–12:7 were tasked with producing 24 SVO structures (both verbally and through graphic symbols) by watching videos of an agent performing a transitive action on a patient. More than half of the responses from the kindergarten-age group did not follow English constituent order; moreover, although the older group performed better than the young group, their results still showed significant differences in English constituent order between verbal and graphic symbol responses. However, it is important to note that recent studies focusing on improving the language outcome of children who use AAC have shown that children with severe speech disorders who received adequate training based on appropriate intervention techniques can easily learn to produced SVO structures and rule-based messages via graphic symbols (Binger et al., 2017, 2020; Soto et al., 2020).

These findings strengthen the notion that atypical structures produced via graphic symbols are related to the graphic symbol modality and not to the child’s linguistic knowledge. Moreover, the superior results produced by using the verbal modality over the graphic symbol modality can be also explained by the general notion that the ability to process external (visual) representation is unlike processing spoken language, a universal ability for all typically developing children (Lee and Karmiloff-Smith, 1996).

The set of graphic symbols used in this study for representing content words (nouns and verbs) included PCS graphic symbols. While this set of symbols is designed to be as transparent as possible, a letter was used for representing the functional word “*and*.” Accordingly, the set of visual forms used in this study vary in their level of iconicity, ranging from transparent to translucent. Previous psycholinguistic studies found that preliterate young children differentiate between drawing and writing, as demonstrated in sorting tasks in which they were asked to decide which combination of elements belongs to a specific notational system. However, in production tasks when they were asked to “write a letter to a friend or “leave a message” their focus remained on the content they wanted to convey, and drawings were therefore used to express that content (Landsmann and Karmiloff-Smith, 1992). This discrepancy is explained by the distinction between notation as a domain of knowledge and notation as a domain referential-communicative tool in which the focus is on the content and usage of drawing. Moreover, Landsmann and Karmiloff-Smith (1992) also explain that one of the distinctions between drawing and writing is the relative closure constraint that is similar to the distinction made in linguistics between open class categories (e.g., nouns and verbs) and closed class categories (e.g., articles and conjunctions); within the open class category, it is always possible to add new elements, while within the closed class the set of elements is finite.

Examination of the sematic-syntactic representation of different syntactic structures in this study revealed that verbal production earned higher scores than graphic production in almost all, but not all, structures. The results revealed an effect of structural complexity and lexicon on graphic symbol production in SVO and coordinated clauses ([SV] AND [SV]) but not in SV structure. This might be explained by SV structure being more directly transmitted onto the graphic symbol modality due to the: (1) Domain of referential-communicative tool: preschoolers prefer to focus on content words (nouns and verbs) represented by iconic symbols to convey their message, and (2) Domain of knowledge: avoidance of the lexicon and syntactic modification of longer and complex structures requires a verb complement and use of a functional word (and) represented by a non-iconic symbol.

### Lexicon and Modality

Children in this study earned higher lexicon scores during verbal production, compared to graphic symbol modality, only in the SVO structure, but not in the SV or SV + SV structures. One explanation for this outcome may be the nature of the task demands. The children were asked to watch a video and verbally express the semantic relation of the verb to its arguments and then to express it via graphic symbols. In the SVO structure, the children needed to identify the relation of the agent (Subject) and the person affected by the action (Object), as well as express the target verb with its two arguments; in the SV and SV + SV structures, they needed to identify the relation of the verb with one argument. As such, SVO structures may lead to less accuracy than SV structures when selecting the target content words (verbs and nouns) via graphic symbols due to lexical voids−missing words in the communication board.

Another explanation may be a strategy of enhancing the communication pace by expressing only specific content words because the video content is known to the child and the examiner and is therefore shared knowledge and common ground. Moreover, some transitive verbs may be less transparent than others, and their meaning dependent on context. For example, the transitive verb HOLD, used in this study as a target verb, was notably a non-transparent graphic symbol, as noted during the familiarization phase. The video presenting the verb HOLD with a boy holding a book was also not clear: seven out of 19 participants verbally indicated the verb READ instead of HOLD (“the boy is reading a book” instead of “the boy is holding a book”). As this answer was not expected, the verb “read” was missing from the communication board, and thus resulted in a lexical void.

### Syntactic Complexity and Modality Use

Children in this study gained higher syntactic scores in SVO and SV + SV, but not in SV, structures. These findings may also be explained by the task demands, as the short constructions of the SV structure may be more easily transmitted onto the graphic symbol modality. These results are similar to the results found English and French, in which structural complexity may play an important role in graphic symbol construction. Short constructions require less modification of spoken constituent order and a lower level of linguistic analysis to complete the task (Trudeau et al., 2007; Sutton et al., 2020).

Furthermore, constructing coordinated clauses may require metalinguistic skills and exposure to formal writing instruction. Representing functional words, such as the conjunction word “*and*,” is challenging as its level of iconicity is very low. Such words are often represented as “sight words” on the communication board; in this study, the word “*and*” was represented by its Hebrew written form. Only 4 (21%) participants identified the letter that represented the word “*and*” when it was first introduced during the familiarization phase. The lack of formal writing instruction and literacy skills among the preschool children affected their ability to transpose the word “*and*” into the graphic symbol modality, resulting in atypical structures of two coordinated clauses, even though they possessed this structure in their mental representation.

### Word Order and Modality

Children in this study earned higher word order scores in all types of clauses (SV, SVO, and SV + SV) using verbal production compared to graphic symbols. As stated before, metalinguistic knowledge and literacy skills may be required to create graphic symbol constructions that maintain the verbal production order. Therefore, it is not surprising that young, typically developing 4- to 5-year-old children did not maintain the word order in graphic symbols. This observation is consistent with graphic symbol findings in other languages that demonstrated metalinguistic skills develop gradually in the early school years and ultimately affect children’s abilities to transmit complex sentences into graphic symbols (Trudeau et al., 2007; Sutton et al., 2020). Moreover, this finding is consistent with those found in English in which typically developing children often produced graphic symbol constructions that did not follow the canonical word order of spoken sentences (Smith, 1996; Trudeau et al., 2010a,b).

### Implications for Clinical Practice

Cross-linguistic studies on graphic symbols are necessary to shed light on the characteristics and constraints of the graphic symbol modality beyond individual differences and linguistic knowledge. Across various languages, including Hebrew, individuals who cannot use their natural speech and who are not yet literate use the same set of graphic symbols (e.g., PCS) to transmit their thoughts and express ambient language. In recent years, there has been impressive progress in designing evidence-based language interventions that enhance the linguistic outcome of individuals who use AAC (Binger et al., 2017; Soto and Clarke, 2017). Moreover, the advanced technology provides access to a wide range of grammatical markers via graphic symbols that support morpho-syntactic representation. As language is developed through language use (Tomasello, 2009), it is essential to provide explicit instruction that supports language growth for individuals who use graphic symbols.

However, constructing the structures of the spoken language via graphic symbols is still a challenge. The question remains to what extent atypical clause structures observed via the graphic symbol modality relate to intrinsic factors of individuals who utilize AAC or the modality itself.

Exploring how typical-developing children who are not yet literate use graphic symbols without adequate training is necessary to understand the relationship between verbal production and the graphic symbol modality. Based on the current findings, professionals working with children aided by AAC as their main modality of communication should take into consideration that atypical construction may not reflect linguistic knowledge. During formal AAC interventions aimed at transmitting spoken language utterances onto the graphic symbol modality, the following should be considered:

(1) Subject verb structures with non-transitive verbs require the least metalinguistic demands and modifications of spoken utterances, specifically when using iconic symbols (e.g., sleep, slide, swing, walk, play).
(2) Subject–verb–object structures impose additional challenges, both in the selection of the graphic symbols that represent the agent who initiates the action and the person affected by the action, and in the ordering of verb arguments in the canonical order. Semantically transitive verbs may be less iconic (such as the verb HOLD) and require further instruction to learn the symbol meaning; and
(3) Constructing two coordinated clauses using the coordination word “*and*” via graphic symbols requires the additional literacy skill of identifying sight words, non-iconic symbols, as well as receiving formal instruction. This is due to the coordination word “*and*” not being iconic and being represented by a sight word.

### Limitations and Future Research Needs

The primary goal of this study was to extend previous findings that graphic symbol construction may differ from verbal utterances regardless of the level of linguistic knowledge of the spoken language and regardless of the specific language. Therefore, the experimental tasks for this study were designed for typically developing Hebrew-speaking children who were not literate but had mastered the syntactic structures used in this study. Various factors may affect the translation of spoken language onto graphic symbols including metalinguistic demands presented by the complexity of the structures, exposure to formal writing, level of symbol iconicity, and symbol availability on the communication board. The children in study received minimal exposure and training in graphic symbol use, and the tasks presented did not examine different metalinguistic knowledge and literacy skills. To generalize these findings and further explore the relationship between verbal and graphic symbol production in Hebrew, additional research is needed- among school-aged children and adults, and with larger samples, longer training periods; and constructions including the use of various functional words. Future research is needed to explore the patterns of non-canonical word order and types of content words that were omitted in the graphic modality.

## Conclusion

The results of this study indicate that typically developing 4–5 year old Hebrew-speaking children display semantic-syntactic representation via graphic symbols that differs from spoken language. Verbal production was superior in lexicality, syntactic complexity, and word order across different task demands presented by different syntactic structures. Differences were notable in structures with transitive verbs (SVO) and two coordinated clauses ([SV] AND [SV]), but not in SV structures. Although preschoolers have the mental representation of these structures, when utilizing the graphic symbols as a referential-communicative tool their focus appears to be on the content they want to transmit, resulting in a focus on content word and iconic symbols. Explicit instruction appears needed to use functional words within clause construction via graphic symbols as these words are represented by non-iconic symbols and relate to the domain of knowledge and do not serve as referential communicative tools.

Similarities between our findings and those in English point to the notion that atypical structures produced via graphic symbols are related to the modality itself and the task demands, not to a child’s linguistic knowledge and specific language. All our conclusions pertain to young children who speak Hebrew; it remains to be seen whether the same relationships hold up across various ages with different clause structures, functional words, literacy and metalinguistic skills.

## Data Availability Statement

The raw data supporting the conclusions of this article will be made available by the authors, without undue reservation.

## Ethics Statement

The study was approved by the IRB of Ariel University (AU-HEA-GH-20190130-1) and was conducted in accordance with appropriate ethical standards. Written informed consent to participate in this study was provided by the participants’ legal guardian/next of kin.

## Author Contributions

GS-H worked on the study design, literature review, data scoring, and writing. LF worked on the study design, data analysis, and writing. All authors listed made a substantial, direct, and intellectual contribution to the work, and approved it for publication.

## Conflict of Interest

The authors declare that the research was conducted in the absence of any commercial or financial relationships that could be construed as a potential conflict of interest.

## Publisher’s Note

All claims expressed in this article are solely those of the authors and do not necessarily represent those of their affiliated organizations, or those of the publisher, the editors and the reviewers. Any product that may be evaluated in this article, or claim that may be made by its manufacturer, is not guaranteed or endorsed by the publisher.

## Footnotes

1 SymbolStix© Available online at: https://www.cricksoft.com/uk/products/symbol-sets

2 Widgit Available online at: https://www.cricksoft.com/uk/products/symbolsets/widgit-symbols.2010.529619
